# Operationalized definition of older adults with high cognitive performance

**DOI:** 10.1590/1980-57642018dn12-030001

**Published:** 2018

**Authors:** Wyllians Vendramini Borelli, Karoline Carvalho Carmona, Adalberto Studart-Neto, Ricardo Nitrini, Paulo Caramelli, Jaderson Costa da Costa

**Affiliations:** 1 Brain Institute of Rio Grande do Sul (BraIns), Pontifical Catholic University of Rio Grande do Sul, Porto Alegre RS, Brazil.; 2 Grupo de Pesquisa em Neurologia Cognitiva e do Comportamento, Faculdade de Medicina de Universidade Federal de Minas Gerais, Belo Horizonte MG, Brazil.; 3 Department of Neurology, Hospital das Clínicas da Faculdade de Medicina da Universidade de São Paulo, São Paulo USP, Brazil.

**Keywords:** memory, aging, neuropsychology, older adults, youthful cognition, memória, envelhecimento, neuropsicologia, idosos, cognição juvenil

## Abstract

Recently, there has been an increasing number of studies on exceptional cognitive aging. Herein, we aim to objectively provide the operationalized characterization of older adults with unusually high memory ability. Some authors have defined them as “SuperAgers”, individuals aged 80 years or older with memory ability similar or superior to middle-aged subjects. On the other hand, the terminology “high-performing older adults” (HPOA) seems to appropriately conceptualize these individuals without exaggeration. A threshold for age is not a reliable criterion, but may be defined as 75 and 80 years of age for developing and developed countries, respectively. We propose that HPOA may exhibit episodic memory test scores equal to or greater than those of individuals aged 50-60 years, according to the validated tables for the respective country. This group must also have global cognition scores within expected average values for age and education. Executive functioning may play a central role in the exceptional memory performance of this group. Further studies are essential to confirm existing findings and may provide important evidence for cognitive aging theory and the neurobiology of dementia.

## RESUMO

O número de estudos sobre envelhecimento cognitivo acima da média vem crescendo recentemente. Neste trabalho, nosso objetivo é fornecer a caracterização operacionalizada de idosos com capacidade de memória excepcionalmente alta. Certos autores definem-nos “Superidosos”, indivíduos com 80 anos ou mais com habilidade de memória similar a adultos de meia-idade. No entanto, a terminologia “idosos de alto desempenho” parece definir de maneira apropriada esses indivíduos, sem restrição excessiva. Apesar de um limite de idade ser imperfeito, ele pode ser definido como 75 ou 80 anos, em países em desenvolvimento ou desenvolvidos, respectivamente. Nós propomos que os idosos de alto desempenho devam ter escores em testes de memória episódica de indivíduos entre 50 a 60 anos, de acordo com tabelas validadas para o país. Esse grupo também deve ter escores de cognição global dentro da média para idade e educação. O funcionamento executivo pode ter um papel central no desempenho excepcional de memória desse grupo. Mais estudos são essenciais para confirmar a existência desses achados e podem fornecer evidência importante para teoria de envelhecimento cognitivo e a neurobiologia das demências.

**Figure 1:**
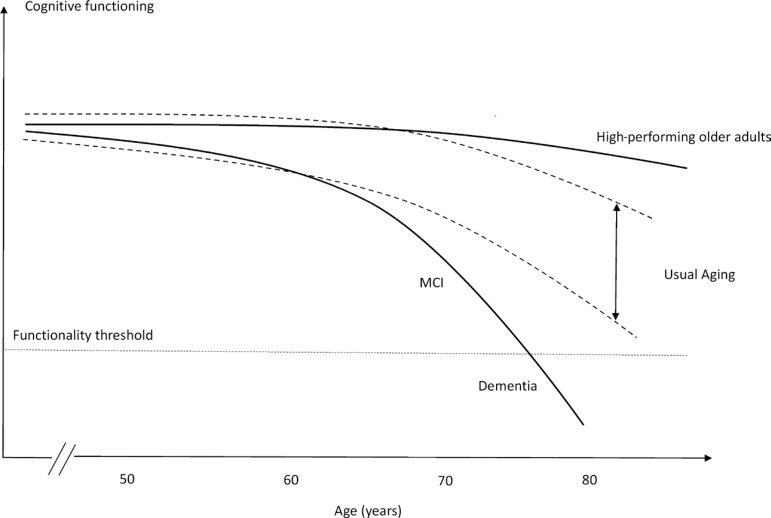
Hypothetical models for different cognitive trajectories of aging.

Aging is a major risk factor for many neurodegenerative disorders, particularly Alzheimer’s disease. Despite the growing effort in uncovering the underlying pathophysiology of this alarming disease, therapeutic failure has been found to be 99% in all clinical trials.^1^ In this context, a different perspective has emerged recently: the study of healthy older adults.^2^ Cognitive performance is a major determinant of healthy aging. It is well established that cognitive decline leads to overall loss of well-being^3^ and functioning in late-life.^4^ It is an independent factor of all-cause mortality^5^^,^ 6 with a massive socioeconomic impact for society^7^ and caregivers.^8^ Rowe and Kahn’s definition of successful aging encompasses a variety of aspects of older adults, including cognition and functioning.^9^^,^ 10 A more recent and specific terminology, in relation to cognition, “successful cognitive aging” was proposed by Depp.^11^ Many cognitive aging theories, such as the concept of cognitive reserve^12^ and brain maintenance,^13^ have emerged in order to explain why some individuals suffer from age-related decline faster than others. Usually, this theoretical model of aging is applicable to high-performing elderly, whose cognitive abilities appear similar to younger individuals (Figure 1).

Notably, these older adults exhibit similar features to middle-aged individuals.^16^ Instead of losing cognitive performance, they seem to be subjected to the underlying mechanisms that minimize the effect of aging, as proposed by the theory of brain maintenance.^13^^,^ 17 Ultimately, this group presents cognitive maintenance as a major characteristic, which consequently leads to independence and well-being. However, there is a lack of consensus in this field and authors have different opinions on defining and conceptualizing individuals with an exceptional cognitive aging trajectory.^13^^-^ 15 Inadequate inclusion criteria may possibly bias the external validity of some studies, such as the selection of middle-aged and older adults in the same group.^18^ Cognitive assessment is also an issue, as different studies were performed using the same terminology but different cognitive tests.^19^^-^ 21 As there is a growing interest in the successful cognitive aging trajectory, these biases may profoundly impact the study of the cognitive aging process and its mechanisms of cognitive maintenance.

In this setting, the aim of the present study was to propose an evidence-based opinion of an operationalized definition of individuals who achieve successful cognitive aging and to determine the inclusion criteria for this group, particularly in developing countries.

## TERMINOLOGY

As described by Depp et al.,^14^ in a review compiling 28 studies, 29 different definitions of successful aging have been proposed so far. As successful cognitive aging is an emerging topic, it has been analyzed from different perspectives leading to numerous different definitions.^19^^,^ 22 ^,^ 23 Nonetheless, it is essential to conceptualize an operationalized, well-defined age and cognitive measure definition before making assumptions about this group of individuals. In addition, a greater variability in cognitive functioning is found in older age than in the younger population, which may potentially be a confounding factor in cognitive aging studies. Thus, there is a need for a single concept applicable to each specific group.

In this context, perhaps the most popular terminology, “SuperAgers”, is defined by individuals aged 80 years or over, with a memory ability similar or superior to middle-aged subjects.^24^ Therefore, non-memory domains were evaluated and participants required to perform, on average, within 1 standard deviation on the Trail Making Test Part B, the 30-item Boston Naming Test and the Category Fluency Test. Individuals were included when they lacked clinical or imaging findings of neurologic or psychiatric conditions and maintained activities of daily living.

Although this definition is useful in defining older adults with exceptional cognitive ability, we believe it to be rather limited. The evaluation of individuals with high performance does not imply only selecting a single class of subjects with a specific neuropsychological criterion. A broader, more comprehensive definition should also include subjects with little or no literacy but with unusual cognitive functioning. Therefore, the definition of SuperAgers might be inadequate for developing countries, due to the high prevalence of low education and its influence on normative values of cognitive tests. Thus, many individuals who are not classified as SuperAgers, but have exceptional memory ability for age and education, might be misclassified as usual agers.

There is a significant effort to elucidate the aging process by studying cognitively average older adults. Selection criteria of many studies includes older adults without signs of cognitive decline.^25^^-^ 27 Nonetheless, these individuals present typical age-related cognitive decline. Here we focus on individuals with cognitive performance that is above average for their age. These individuals potentially have maintained their memory ability throughout the aging process, scoring similar to middle-aged subjects.

We propose a general nomenclature for individuals who achieve successful cognitive aging. The term “High-performing older adults” (HPOA), first cited by Cabeza et al.,^19^ provides an expanded concept of individuals who maintain unusually good cognition. Although some studies have used the same terminology, their selection criteria were not the same. While some studies used memory scores for defining HPOA,^16^^,^ 19 ^,^ 24 others used educational level or non-memory domains.^20^^,^ 21 ^,^ 28 ^,^ 29 The heterogeneity of previous studies supports the need for a single terminology and evidence-based criteria for classification of HPOA. Imperative for this definition, we propose two main features for classifying an individual as HPOA, namely suitable age threshold and adequate cognitive measurements, either of memory or non-memory domains.

## AGE CUT-OFF

An adequate age criterion is vital for proper selection of older individuals with high cognitive performance. Different age criteria are used by different authors, ranging from 60 to 80 years.^16^^,^ 22 ^,^ 30 ^,^ 31 Since this limit appears to vary substantially across studies, we suggest that a single age limit for defining HPOA can benefit further generalization of results.

The concept of aging is cultural and dynamic. The onset of old age is a matter of discussion, with a massive impact on public health policies, labor force, and retirement. Conventionally, aging is arbitrarily determined as occurring at 65 and 60 years of age in developed and developing countries, respectively.^32^^,^ 33 The World Health Organization (WHO) and the Brazilian Elderly Statute use the standard of age 60 to describe older adults, but the WHO clarifies that this is not a precise cut-off.^34^ Moreover, aging is directly affected by life expectancy, which plays a major role in this definition. Individuals born in resource-rich countries have a higher possibility of attaining an older age. An individual born in the United States, for both sexes, lives almost five years longer when compared to a Brazilian subject (79.3 vs. 75).^35^

Age-related cognitive decline usually intensifies at around 60-65 years of age^13^ and should be carefully addressed in cognitive aging studies. Longitudinal and cross-sectional samples indicate that between 20-60 years there is little or no cognitive decline,^36^^,^ 37 but it is marked after the age of 74 years.^38^ Age-related brain atrophy follows a non-linear process, in which prefrontal and parietal regions are more vulnerable.^39^^-^ 41 Grey matter density of cognitively normal adults also undergoes more rapid decline from 7 to 60 years, with lower atrophy rates thereafter.^42^ Studies that include individuals aged 60 to 65 years may introduce a bias, as age-related cognitive decline is already underway. Hence, it is believed that individuals who do not suffer from age-related cognitive decline up until 75 years of age have a higher likelihood of maintaining their cognitive ability, given that they do not have neurological diseases causing cognitive impairment or dementia.

The HPOA age cut-off may vary according to sociodemographic and cultural characteristics. We propose a threshold of successful cognitive aging according to sociocultural characteristics of the studied sample. An adequate, though imperfect, cut-off could be determined for the developed and developing countries as 80 and 75 years, respectively. These proposed cut-offs account for the 5-year difference both in life expectancy and in the definition of older adults between developed and developing countries, as previously cited in this topic. Furthermore, developing countries have lower educational levels and higher social discrepancy than developed countries. Thus, a higher age threshold may incorrectly select individuals with a better educational and economic background in these nations.^43^^-^ 45

## COGNITIVE CRITERIA

### Memory scores

The extent of usual, age-related cognitive decline implies a subject’s ability for independence and functioning in daily life. The processes of memory encoding, storage, and retrieval decline over the course of the whole lifetime, where encoding is particularly vulnerable to aging and seems to decline across the lifespan.^36^ The encoding of novel memories is a major hippocampal function and shows a strong relationship with memory impairment in AD.^46^^-^ 48

Several studies regarding high performance in old age rely upon episodic memory evaluation as a major defining factor.^16^^,^ 22 ^,^ 23 ^,^ 49 In agreement with other studies in this field, the definition of HPOA must consider episodic memory performance as essential because of its clear relationship with cognitive impairment and dementia. Besides, the biological mechanisms of HPOA may differ to that of AD-type dementia given their contrasting clinical features. A cognitive composite of executive functions, memory, and global cognition reliably determines the first signs of AD,^50^ which highlights the importance of these functions in this condition.

There are several validated and reliable tools for episodic memory measurement in the English language. Specifically, psychometric properties for Brazilian neuropsychological tests lack diversity and adequacy of data. The Rey Auditory-Verbal Learning Test (RAVLT), typically applied in older adults, reliably distinguished not only high-performing groups from controls^51^ but also different age groups.^52^^,^ 53 This tool also showed good replicability in distinguishing normal from pathologic cognitive decline with aging.^54^ However, the Brazilian population tends to be penalized due to lack of suitable scores for low levels of education on the RAVLT.^52^ In this context, visual memory tasks may be useful, such as the visual task of the Brief Cognitive Screening Battery^55^ and the Rey-Osterrieth Complex Figure.^56^ Visual memory tasks have shown little association with the educational level of individuals,^57^^,^ 58 while reliably measuring their memory performance.

There are many factors that must be considered when choosing the adequate neuropsychological test to measure episodic memory capacity. The major factor in considering any test is the development of normative data for the studied population. There are many suitable tests developed specifically for measuring verbal memory (Table 1), such as the California Verbal Learning Test^59^ (not available for the Brazilian population) and the Free and Cued Selective Reminding Test,^60^ for example. Some tests are commonly used in Brazil, but their references are standardized scores validated for the North American population, such as the Wechsler Memory Scales.^61^

**Table 1:** Commonly used memory tests for the Brazilian population.

Cognitive test	Category evaluated
Rey Auditory-Verbal Learning Test	Verbal
Free and Cued Selective Reminding Test (free delayed-recall)	Verbal
Logical Memory Immediate and Delayed Recall (WMS III)	Verbal
California Verbal Learning Test*	Verbal
Hopkins verbal learning test	Verbal
Rey-Osterrieth Complex figure	Visual
Visual Memory Index (WMS III)	Visual
Brief Cognitive Screening Battery	Visual

* Normative data not available for the Brazilian population.

As defined in previous studies, HPOA must represent the highest level of memory performance in elderly. Some studies define the top 20% of the sample as high memory performers, which may introduce a bias. This method selects only high performers in a single sample, which may not adequately represent the high-performing individuals in the general population as a whole.

Nonetheless, the rigid memory score definition used by Rogalski et al.,^24^ greater than or equal to the normative values of individuals at 50-60 years, is adequate for several reasons. First, this age range considers the memory scores of individuals before the typical age-related memory decline, as described in Topic 2. Second, as it is related to the test, this method of selecting HPOA is independent of sample size. Third, it facilitates the comparison of data with studies from other contexts and cultures that also define HPOA as older adults scoring greater than or equal to individuals of 50-60 years. Thus, we propose that HPOA may be selected with a memory test score that is equal to or above normative values of individuals at 50-60 years of age, but not necessarily using the RAVLT. Individuals with low educational level may be evaluated with visual memory instead of verbal memory tests (Table 1).

### Non-memory scores

Global cognition must also be evaluated in order to successfully select individuals with excellent memory performance. A low score on this type of test may indicate a subclinical pathologic process that does not affect the memory system. On the other hand, above average scores suggest that these individuals have an optimal performance when associated with unusually high memory scores. It is important to note that the cognitive evaluation typically performed in a neurological or psychiatric routine is essentially not a measure of global cognition. Some tools for dementia screening are excellent for the diagnosis of mild cognitive impairment or dementia staging but should be avoided as classifiers for “cognitively normal”, such as the Mini-Mental State Examination (MMSE) or the Montreal Cognitive Assessment (MoCA).^62^ A comprehensive and validated tool may be used for classifying subjects, such as the Addenbrooke’s Cognitive Evaluation-Revised or the Mattis Dementia Rating Scale.^63^^,^ 64

Frontal lobe functioning seems to be particularly vulnerable to aging.^27^^,^ 36 Previous studies indicate that attention is essential for memory formation and precedes memory consolidation.^65^^-^ 67 Furthermore, inhibitory control, a component of the attentional system, shows an exacerbated decrease after 60 years of age,^68^ although this does not affect selective attention.^69^ The frontostriatal system may be the major cause of decline in executive functioning in nondemented individuals; this has an indirect but strong impact on memory encoding.^36^^,^ 70 White matter also seems to be preferentially weakened in anterior tracts.^71^^,^ 72 Executive functioning in HPOA is still open to debate, as a recent study suggests that a group of cognitive maintainers may rely upon frontal mechanisms for memory maintenance.^22^ Accordingly, the anterior cingulate is associated with the cognitive control and regulation of attention,^73^^-^ 75 which corroborates that it may play a key role in the exceptional memory in HPOA.^16^^,^ 22

Processing speed is a strong indicator of age changes in memory ability^76^ and also seems to be associated with HPOA.^22^ Some functions appear to be less vulnerable to age-related cognitive decline, such as semantic memory, vocabulary, and fluency skills. Both are linked as crystallized intelligence and tend to enhance with aging.^27^^,^ 77 ^,^ 78 However, it is believed that these functions are not central to cognitive maintenance in aging.

## UNANSWERED QUESTIONS AND LIMITATIONS

A complete understanding of the mechanisms at play in older adults who successfully preserve their memory may reveal key processes in cognitive maintenance. There is still a lack of knowledge in understanding usual and unusual aging. Some recent studies have focused on preclinical stages of Alzheimer’s disease in order to discover early biomarkers of memory decline. The promotion of successful cognitive aging holds great promise in targeting biomarkers, potentially improving cognitive function in later life.

The frontal lobe has been shown to be particularly vulnerable to aging and neurodegenerative processes.^36^ Only one study evaluated executive functioning in HPOA and showed an intriguing preservation of these functions.^22^ Further, subdomains of attention have not been explored in HPOA to date. Further investigations involving frontal assessment tests are needed to better elucidate the role of executive functions and processing in HPOA.

Some neuropsychological tests are not available in many languages. There is a preference for the evaluation of specific cognitive domains in clinical practice, which may imply a misuse of non-culturally adapted versions of tools. Thus, translation, validation, and development of normative values are needed in order to reliably measure the cognitive functioning of older adults, which must be based on cultural and demographic factors.

There is still a need for longitudinal studies on HPOA. Individuals with high cognitive performance may have progressive decline, which is not measurable using a cross-sectional design. In addition, it is not yet known if HPOA cumulate a higher cognitive reserve than usual agers or its relationship to brain maintenance. Only by carefully addressing studies on cognitive aging, will we be able to advance in distinguishing preclinical AD from usual cognitive aging.

## CONCLUSION

In summary, in this study we proposed an evidence-based selection criteria for older adults with exceptional cognitive performance. This study plays a role in defining these individuals according to age and cognitive criteria (Table 2), in contrast with the heterogeneity of studies using different measures and terminologies. With a single concept of HPOA, this study may provide support to better understand the cognitive aging process.

**Table 2:** Operationalized definition of High-performing older adults.

	Age	Memory test scores	Non-memory test scores
Developed countries	≥ 80	Aged ≥50-65 years	Within expected average for age and education
Developing countries	≥ 75		
